# Lifetime existence of a core of mutualistic symbionts and functionally uncoupled taxa in the gut of a Mediterranean cohort

**DOI:** 10.1038/s41598-026-35033-3

**Published:** 2026-01-09

**Authors:** Susana Ruiz-Ruiz, Samuel Piquer-Esteban, Benjamí Pérez-Rocher, Vicente Pérez-Brocal, Vicente Arnau, Alejandro Artacho, Wladimiro Diaz, Nuria Jiménez-Hernández, Javier Pons, José A. Castro, Andrés Moya

**Affiliations:** 1 Area of Genomics and Health, Foundation for the Promotion of Sanitary and Biomedical Research of Valencia Region (FISABIO-Public Health), Valencia, 46020 Spain; 2 Biomedical Research Networking Centre for Epidemiology and Public Health (CIBERESP), Madrid, 28029 Spain; 3 Institute for Integrative Systems Biology (I2SysBio), University of Valencia and Spanish National Research Council, Valencia, Spain; 4 Department of Biology, University of Balearic Islands, Palma de Mallorca, 07122 Spain

**Keywords:** Microbiota throughout life, 16S rRNA gene, Metagenomics, Metatranscriptomics, Stability, Core, Computational biology and bioinformatics, Microbiology

## Abstract

While a proportion of the microbiota plays a beneficial role, there is no conclusive evidence that the entire microbiome is mutualistic. Here, we have studied the intestinal microbiota of three healthy age groups from the Valencian Region (Spain). We have periodically obtained stool samples to determine the 16S rRNA gene amplicons, metagenomes, and metatranscriptomes, and we have observed that the microbiota’s stability differs with age, being less stable in infants. Regarding analyses of the conserved microbiota across the three age groups throughout the study period, shared genera account for about 60%. In addition, we identified a core of microbial taxa present in all individuals, which could represent mutualistic symbionts. Finally, in a previous study, we detected that tryptophan and indole production by intestinal bacteria decreases substantially with host age. Metagenomics and metatranscriptomics analyses show that tryptophanase mRNA synthesis in the genus *Akkermansia* is approximately 10 times lower in adults and the elderly than in children, consistent with this enzyme’s low levels or absence in these groups. Consequently, this supports the hypothesis that an uncoupling might occur between some microbiota taxa and the human host at older ages.

**Supplementary Information:**

The online version contains supplementary material available at 10.1038/s41598-026-35033-3.

## Introduction

The human microbiota is the set of microorganisms found in the host at a given point in time or throughout its lifetime. The microbiome is the subset living in symbiosis with the host for all or a substantial part of their lifetime. The mutualistic microbiome is the subset of the microbiome that lives in mutualistic symbiosis with the human species^1^. The human gut microbiota influences the host’s health, playing a crucial role in immune system development, infection prevention, nutrient acquisition, and, probably, the proper functioning of the brain and nervous system^2,3^. The microbiota is causal in the development of pathologies in animal models of human diseases, such as obesity and autoimmune and neurological disorders^4–6^. Causality is more challenging to establish in humans. However, environmental factors that alter the development of the microbiota are associated with disease risk^7,8^, such as the type of birth^9^, breast milk versus artificial formula^10^, diet^11–13^, or treatment with antibiotics at an early age^14^. Dysbiosis of the intestinal microbiota involves significant alterations in its composition and function compared with healthy controls^15,16^. This alteration may differ depending on the studied omic level^17^. Notably, authors studying pathologies have reported that healthy controls generally carry an associated microbiota (healthy microbiota) that differs from the dysbiotic microbiota of hosts suffering from disease^16^. However, it is unclear for many diseases whether dysbiosis is a cause or a consequence^5^.

All this complexity makes it rather challenging to define what constitutes a healthy microbiota^2^. In this respect, longitudinal studies of the gut microbiota help to differentiate dysbiotic alterations from those that are not, as well as significant non-dysbiotic changes between life periods or time intervals within ages^18^, in response to specific environmental, genetic, geographical, or other factors^19,20^. Under the umbrella of the USA’s Integrative Human Microbiome Project, the first longitudinal works with large cohorts have recently become available, revealing that the microbiota has detectable effects on several illnesses, such as inflammatory bowel diseases^21^, on pregnancy and preterm birth^22^, or stressors affecting individuals with pre-diabetes^23^.

Some longitudinal studies on healthy microbiota have assessed the dynamics and stability of the microbiota at specific ages, for example, in children and pubertal individuals^24,25^, adults^26,27^, and the elderly^28^. They try to identify a core of invariant taxa with a presence above a certain threshold or, more interestingly, the minimal microbiota fraction maintained over increasing time intervals. At least for adults, about 70% of taxa (species and even strains) remain stable for a year, with few changes after 4 years^26,27^. However, few studies aim to evaluate microbiota dynamics throughout life, identify microbiota interactions with physiological aging processes, or develop microbiota-based health surveillance for older adults^29^.

In the present work, we characterized the composition and function of the gut microbiota of a healthy Mediterranean cohort, classified into three age groups, Infants, Adults, and Elderly (the IAE cohort), followed up regularly for over two years, to detect the existence of a set of mutualistic symbionts constituting the microbial core of the Mediterranean cohort throughout their entire lifespan and to evaluate the stability, robustness, and buffering of the microbiota at different age periods.

## Results

**Fig. 1:**
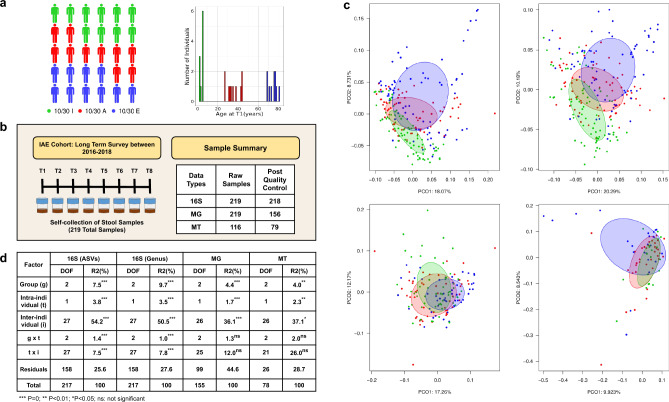
Multi-omics of fecal samples in the IAE cohort study. (**a**) Thirty healthy participants from the Valencian Region (Spain), with an age distribution spanning groups I, A, and E, were recruited to characterize microbiome communities throughout life using 16S, MG, and MT. (**b**) A long-term survey was conducted in which up to eight fecal samples per individual were attempted to be collected. This yielded 219 MG and 116 MT, which, after quality control, resulted in 218 16S rRNA gene amplicons, 156 whole-genome shotgun MG, and 79 MT. (**c**) PCoA with Bray–Curtis dissimilarities for all samples based on 16S rRNA gene taxonomy (above from left to right, ASVs and genus levels, respectively) and functions (bottom from left to right, MG, and MT, respectively). (**d**) Summary of the nested Permanovas, where the *g, t,* and *i* interactions were estimated for taxonomy and functions. DOF indicates degrees of freedom, and R2(%) represents the percentage of explained variance by each factor and interaction. Stars correspond to the statistical significance of the tests.

The IAE cohort was classified into three age groups (Fig. 1a; Supplementary Table 1). Fecal samples were obtained from each individual at different times over a maximum period of 723, 754, and 524 days for Infants (group I), Adults (group A), and Elderly (group E), respectively (Supplementary Table 2) and with average sampling periods of I = 517.3 ± 189.5, A = 499.4 ± 214.3 and E = 362.6 ± 142.8 days (mean ± sd). Of the 219 samples collected, 13 were reported to have antibiotic profilaxis (mostly in infants) and six intestinal episodes. Fecal samples were subjected to 16S and whole genome shotgun metagenomics (MG) and metatranscriptomics (MT). 218 samples for the 16S data (I = 74, A = 70 and E = 74) and 156 for MG (I = 55, A = 51 and E = 50) passed quality controls. MT was also measured at three or more time points per subject, with some exceptions in which only one or two could be determined. This resulted in 116 samples, of which 79 passed quality controls (I = 19, A = 36, and E = 24) and were used for downstream analyses (Fig. 1b). A PCoA based on the Bray–Curtis dissimilarity of the four datasets – 16S ASVs and genus taxonomy, MG and MT functions (Fig. 1c) showed a high heterogeneity, with a variation explained by the first two axes not higher than 30.47%, attributable to a high inter-individual variation. To test the effect of inter-individual variation and the contribution of two additional factors (age group and intra-individual variation), we conducted a nested Permanova (Fig. 1d). Our 16S data, MG, and MT datasets yielded significant effects. Although the higher percentage of variation was due to inter-individual differences (*i*), significant effects of age group (*g*) and intra-individual differences (*t*) were also observed. Noteworthy was the significant effect of *g* × *t* and *t* × *i* in the 16S dataset, indicating that age groups differ in their responses to intra- and inter-individual variation. The latter effect was not detected with the MG or MT dataset (Fig. 1d). CCA and Adonis tests also showed significant differences in taxonomy (ASVs and genus levels) and functions (MG and MT) across age groups (Supplementary Fig. 1).

### Differences in taxa between age groups

The distribution of relative abundances (Supplementary Fig. 2) showed that the fecal microbiota in the aggregated taxa for the three age groups was dominated by phyla Firmicutes and Bacteroidota (collectively, I = 73.9%, A = 78.9% and E = 76.5%), which increased with age for Bacteroidota (I = 22.5%, A = 24.6% and E = 25.4%), followed by Actinobacteriota, Verrucomicrobiota, and Proteobacteria (collectively I = 14.9%, A = 9.6% and E = 7.9%), which decreased with age for Actinobacteriota (I = 12.2%, A = 8.6% and E = 6.6%). At the genus level, the 20 most abundant taxa contributed 78.9% to Infants, 78.6% to Adults, and 73.2% to Elders. Nine genera belonged to class *Clostridia* (*Faecalibacterium*, *Ruminococcus*, *Subdoligranulum*, *Anaerostipes*, *Coprococcus*, UCG-002, unclassified members of families of *Eubacterium coprostanoligenes* and *Lachnospiraceae*, as well as, unclassified members of *Clostridia* itself), five to *Bacteroidia* (*Bacteroides*, *Prevotella*, *Prevotellaceae* NK3B31 group, *Alistipes*, *Parabacteroides*), one to *Bacilli* (*Streptococcus*), two to *Negativicutes* (*Phascolarctobacterium* and *Dialister*), and three others, *Bifidobacterium, Collinsella,* and *Akkermansia* to classes *Actinobacteria*, *Coriobacteriia,* and *Verrucomicrobiae*, respectively. On the other hand, the Chao 1 richness estimator and Shannon diversity index increased significantly with age (Supplementary Fig. 3).

**Fig. 2:**
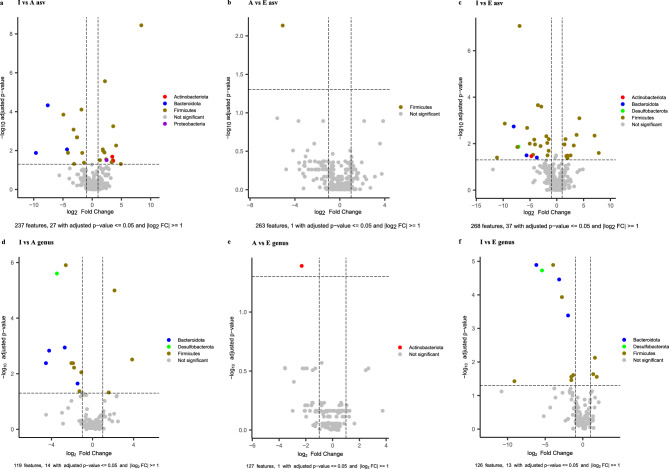
Volcano plots for differential taxonomy analysis based on the 16S rRNA gene. ANCOMBC was used to perform differential analysis comparisons between age groups at the ASVs (**a-c**) and genus (**d-f**) taxonomic levels. Significance was set with adjusted p − value <  = 0.05 and |log2 FC|> = 1. Adjusted p-values were corrected with the Benjamini–Hochberg method.

Analyses of ASVs (Fig. 2a-c) and genera (Fig. 2d-f) showed significant differences between age groups for different taxa (Supplementary Table 3 for further details). Taxa differentially abundant between groups I-A comprised 27 ASVs and 14 genera. Regarding groups I-E, 39 ASVs and 13 genera showed significant differences. Finally, 1 ASV and one genus (*Rothia*) showed significant differences between groups A-E. Genera such as *Intestinibacter* and *Faecalitalea* decreased significantly from Infants to Elders, while *Blautia, Butyricimonas,* and *Coprobacter* increased significantly with age. Others, such as *Veillonella*, and *Intestinibacter,* decreased significantly from group I to A.

### Analysis of the core microbiota

**Fig. 3:**
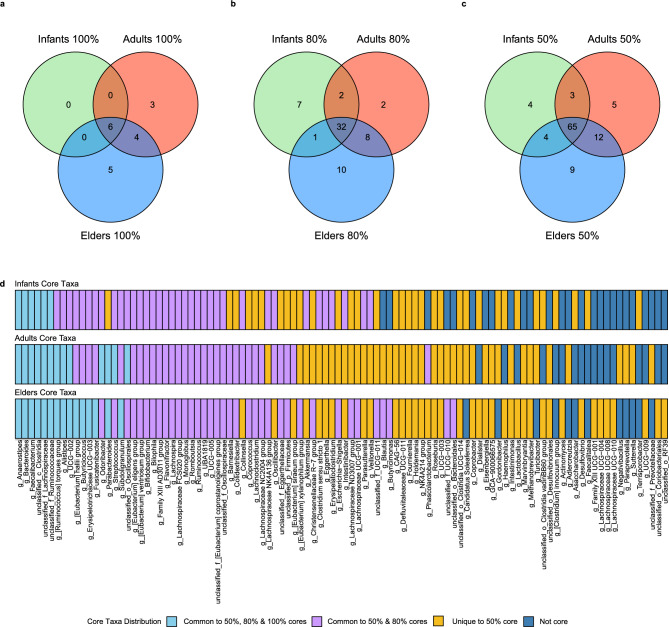
Core microbiome analysis. Various cores were defined at the genus level based on 16S rRNA gene prevalence at high (**a**), medium (**b**), and low (**c**) levels for the different age groups, with presence in at least 100%, 80%, and 50% of the samples per age group, respectively. Core taxa distributions between age groups are also represented (**d**).

Only genera were detected in 100%, 80%, and 50% of the samples across all time points and age groups (Supplementary Table 4; Fig. 3). The 100% core microbiota (Fig. 3a) consisted of 18 genera in all samples in at least one age group. Of those, only six genera (*Anaerostipes, Faecalibacterium, Bacteroides*, two non-characterized members of families *Lachnospiraceae* and *Ruminococcaceae*, and one non-characterized member of class *Clostridia*) were shared by all age groups. Furthermore, *Faecalibacterium*, *Bacteroides*, and unclassified members of family *Lachnospiraceae* were among the most abundant taxa for the different age groups and individuals (Supplementary Fig. 4a-b). For the remaining 12 genera, four were shared by Adults and Elders (*Alistipes*, [*Ruminococcus*] *torques* group, UCG − 002, and *Parabacteroides*), three were only identified in Adults (*Streptococcus*, *Odoribacter*, and a non-characterized member of order *Oscillospirales*), and five only in Elders (*Butyricicoccus, Erysipelotrichaceae* UCG − 003, [*Eubacterium*] *hallii* group, *Subdoligranulum,* and *Fusicatenibacter*). The 80% core microbiota (Fig. 3b) showed more core genera (62), 32 of which were shared simultaneously by all age groups. Among these, we found five taxa that, while falling short of the high core threshold, had prevalence above 90% in all age groups (*Bifidobacterium*, [*Eubacterium*] *eligens* group, Family XIII AD3011 group, unclassified members of the [*Eubacterium*] *coprostanoligenes* group, and *Oscillospiraceae*). Furthermore, *Bifidobacterium* was among the most abundant taxa across age groups and individuals (Supplementary Fig. 4a-c). Conversely, pairs of age groups shared 11 genera (I-E = 1, I-A = 2, and A-E = 8), while 19 cores were unique to a single age group (I = 7, A = 2, and E = 10). Finally, for the 50% core microbiota (Fig. 3c), more genera were above this cut-off (102), with 65 overlapping all age groups; pairs of age groups shared 19 taxa (I-E = 4, I-A = 3, and A-E = 12). In contrast, 18 core taxa were unique to a single age group (I = 4, A = 5, and E = 9). Furthermore, the number of core taxa increases with age independently of the threshold used (I 100% = 6, 80% = 42, 50% = 76; A 100% = 13, 80% = 44, 50% = 85; E 100% = 15, 80% = 51, 50% = 90). Moreover, changes in prevalence and detection with age are also observed (Supplementary Fig. 4c-d). Some core taxa show a consistent increase in prevalence with age (*Blautia*, *Butyricimonas*, *Desulfovibrio,* or *Coprobacter*). In contrast, others show a decrease in prevalence and even become non-core taxa (*Faecalitalea*, *Gordonibacter*, *Terrisporobacter*, or *Turicibacter*).

### Taxa conservation across time within age groups and throughout life

**Fig. 4:**
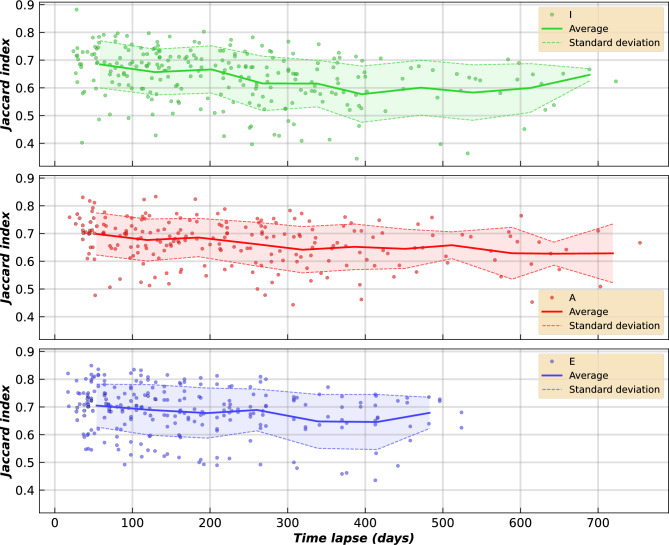
Individuals’ Jaccard similarity indexes at the genus level based on 16S rRNA gene. Only values calculated as a function of the time lapse between samples of the same individuals for the different age groups are presented. Values between individuals were not calculated. Means and standard deviations were computed using 60-day time bins.

The Jaccard similarity index at the genus level (Fig. 4) showed a general decrease in shared genera with increasing time intervals, with about 60% across Infants, Adults, and Elderly. However, with increasing time intervals, the decrease is generally lower in Adults, especially in Elders, than in Infants.

**Fig. 5:**
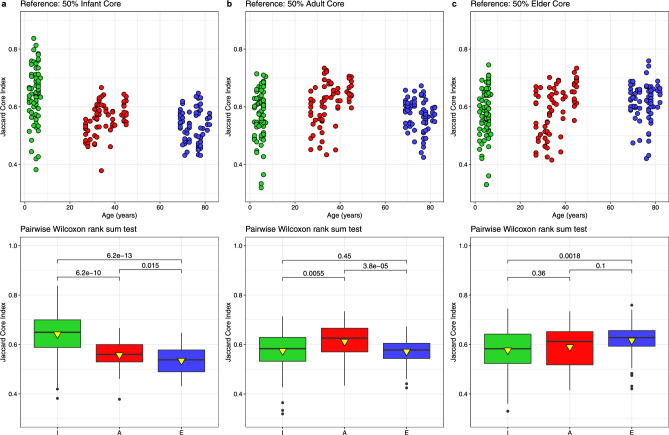
Jaccard core indexes at the genus level based on the 16S rRNA gene. Jaccard similarity indexes were calculated taking as a fixed reference the 50% prevalence core taxa for the Infants (**a**), Adults (**b**), and Elders (**c**) age groups. Top panels present the Jaccard core indexes as a function of age, while the bottom boxplot panels represent comparisons between age groups using the Wilcoxon rank sum test. In the boxplots, the black line within the box marks the median, and the yellow triangle the mean. Significance was set with p − value <  = 0.05.

It is of interest to identify the shared taxa throughout an individual’s entire lifespan and measure the corresponding degree of decay in the Jaccard index. Since we do not have information on the gut microbiota of a healthy individual throughout their lifespan, we applied a modification of the Jaccard index (see Methods). In this case, Jaccard indices were calculated, taking as a fixed reference the 50% core taxa for Infants, Adults, and Elders, as a way to study how the conservation of the microbiome changes over time for each sample with respect to the expected microbiota in at least half of the samples of each age group. The time interval ranged from 2 to 82 years, representing a maximum difference of 80 years between Infants and Elders of the IAE cohort. When comparing the different samples with the Infant core microbiome (Fig. 5a), we observed a steeper decline in similarity with age, with significant differences across all age-group combinations. On comparing the different samples with the core microbiome of Adults (Fig. 5b), similar values were observed, except for Adults, which showed values significantly higher than those in the other age groups. In contrast, compared with the core microbiome of Elders (Fig. 5c), a slight increase in similarity with age was observed, significant only between Infants and Elders.

### Differential functional analyses of MG and MT

**Fig. 6:**
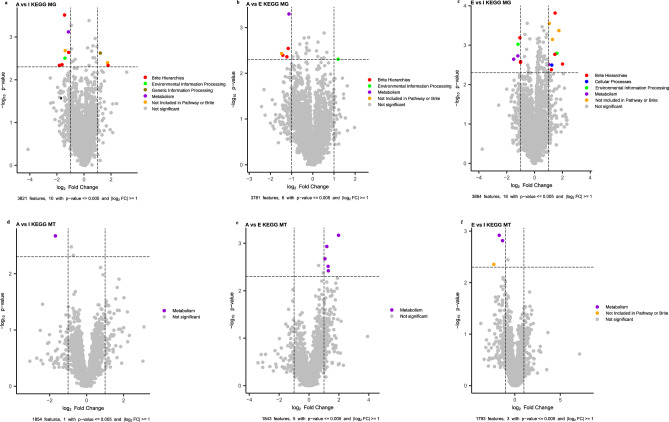
Volcano plots for differential functions analysis. A linear mixed model implemented in the R library nlme was used to test differential expression between age groups for MG (**a-c**) and MT (**d-f**). Significance was set with p − value <  = 0.05 and |log 2 FC|> = 1.

Both MG (Fig. 6a-c) and MT-based analyses (Fig. 6d-f) showed a relatively lower number of significant differences between groups I-A (MG: 10 functions and 12 pathways; and MT: 1 and 4, respectively), groups A-E (MG: 6 functions and 18 pathways; MT: 5 and 17, respectively), and the most extreme age groups I-E (MG: 16 functions and 30 pathways; MT: 3 and 5, respectively). See Supplementary Table 5 and Supplementary Table 6 for further details.

It is of interest to mention some of the pathways that showed significant differences between groups in MG: flavone and flavonol biosynthesis (K000944), and linoleic acid metabolism (K000591), being significantly higher in Infants than in Adults and ascorbate and aldarate metabolism (K000053) being significantly higher in Infants than in Elders while the rheumatoid arthritis pathway (K005323) was significantly higher in Adults than in Infants and Alzheimer disease (K005010) and epithelial cell signaling in the *Helicobacter pylori* infection pathway (K005120) were significantly higher in Elders than in Infants, among others. On the other hand, secondary bile acid biosynthesis (K000121) increased significantly with age when compared to Infants. Furthermore, glycosaminoglycan degradation (K000531) decreased with age, approaching significance between Infants and Elders. Flavonoids, which are subject to gut bacterial transformation, primarily contribute to lower cardiovascular disease and cancer-related mortality^30^. In contrast, host colonic glycans, including glycosaminoglycans, are catabolized by the microbiota to produce short-chain fatty acids, thereby improving human health^31^. Furthermore, a considerable body of evidence suggests that the gut microbiota is tightly linked to the aging process of its host^32^. In MT, it should be noted that the longevity-regulating pathway (K004213), arachidonic acid metabolism (K000590), and taurine and hypotaurine metabolism (K000430) were significantly higher in Infants than in Elders.

### Taxonomic assignments to bacterial protein and age-associated metabolite deficits

In a previous study^33^, we showed that in the proteome of the three age groups of the IAE cohort, at least two KOs, the K01667 (TnaA; tryptophanase) and K01696 (TrpB; tryptophan synthase) were significantly higher in Infants compared to Adults, and were below the detection limit in Elders. TnaA and TrpB catalyze the final steps in tryptophan biosynthesis and its further metabolism into indole, respectively. However, are the taxa involved in producing those proteins inactive or absent in elderly individuals?

**Fig. 7:**
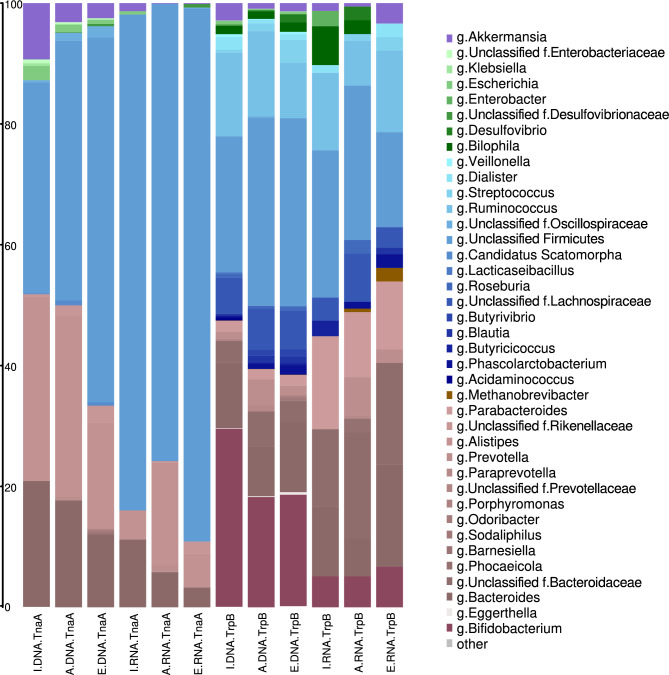
TnaA and TrpB producing taxa. The average composition per age group of TnaA (K01667) and TrpB (K01696) producing taxa is shown for whole genome shotgun MG and MT, indicated as DNA and RNA, respectively.

In the case of the TnaA protein, MG data have shown differences in the taxonomic composition responsible for synthesizing this enzyme across age groups (Fig. 7). The number of MG-reads assigned to K01667 was 5,412 for Infants, 2,831 for Adults, and 4,279 for Elders. The MT data also showed inter-group differences in which taxa express the genes that would code for this enzyme. Interestingly, the number of assigned MT reads to K01667 (I = 374, A = 1,020, and E = 689) decreased sharply compared to those of the MG. Most of them could not be assigned to any taxon at the genus level (I = 82.1%, A = 75.9% and E = 90.9%). The number of MT sequences detected in Infants compared to Adults and Elders is striking, given that the previous levels of the protein/enzyme TnaA and the indole metabolite were significantly higher in Infants than in the other two, where they were practically undetectable^33^. Regarding the previous question, we suggest that in the case of the TnaA protein, a detailed examination shows that mRNA synthesis of TnaA by the genus *Akkermansia* is around ten times lower in Adults (13.4 times lower) and the Elderly (8.9 times lower) than in Infants (I = 1.34%, A = 0.1% and E = 0.15%), aligning with the low prevalence or absence of this enzyme in the A and E groups.

In the case of TrpB, the MG data also showed differences between age groups (Fig. 7), with the number of reads assigned to K01696 being 14,045 for Infants, 10,882 for Adults, and 7,608 for Elders, a number much higher than for enzyme TnaA. Regarding the MT data, inter-group differences were also observed. The number of MT reads assigned to K01667 (I = 78, A = 176, and E = 89) decreased sharply compared to those of the MG, being practically undetectable. The mRNA synthesis for TrpB shows that the genus *Bilophila* was aproximately 3 times lower in Adults than in Infants and was not detected in Elders (I = 6.41%, A = 2.27%, and E = 0%). The other two genera, *Enterobacter* and *Butyricicoccus*, were only present in Infants, consistent with the low prevalence or absence of this enzyme in groups A and E.

### Stability analysis

As previously stated, it is possible to compare cohorts of individuals corresponding to well-defined life periods^25^. We applied the stability analyses developed^34^ based on Taylor’s power law to determine and compare the microbiota stability of the three age groups, using the relative composition of genera at different time points across the individuals comprising our cohort. The main parameters of the model are V, the amplitude of the fluctuation that estimates the variance in the abundance of each taxon, and β, the power-law index (see methods).

**Fig. 8:**
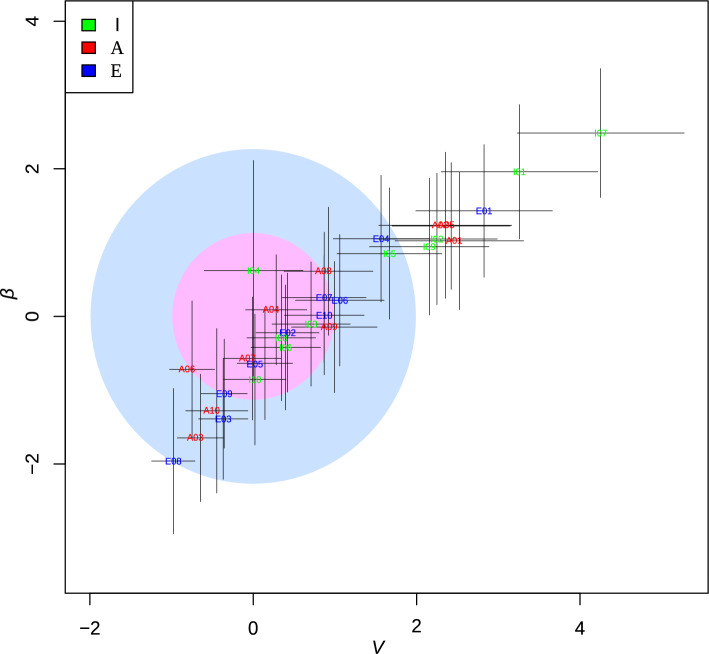
Taylor’s law parameter space at the genus level based on the 16S rRNA gene. The large inner-pink circle corresponds to the 68% confidence level region of the reference subjects (adults) in Taylor’s parameter space, while the outer-blue circle delimits the 98% confidence level region. Lines represent standard errors that place the gut microbiome in Taylor’s parameter space for each subject. Parameters were standardized (standard deviation units) to the reference group (A group).

In our study, the fit to the power law was always robust (R^2^ > 0.894 for all individuals except I04, with only two time points, R^2^ = 0.785; Supplementary Table 7 for further details) and did not depend on microbiome conditions. *β* was always less than 1 across all individuals, regardless of age group. This would indicate that the most abundant genus in the microbial community was less susceptible to perturbations than the other genera. Interestingly, however, *V* appears to vary across age groups, being higher on average in group I (0.328) than in groups A (0.272) and E (0.259). It is worth noting that more Infants were outside the healthy window than members of the Adult or Elderly groups (Fig. 8).

### Robustness analyses of taxa and functions

As expected, because attenuation and buffering were calculated from the abundances of each 16S ASV in each sample (Supplementary Table 8), they varied between individuals and samples across all age groups (Supplementary Fig. 5). For attenuation, this variation ranged similarly across age groups, whereas buffering showed differences and was more variable in Elders than in Infants or Adults. Furthermore, buffering was significantly lower in Adults than in the other two age groups.

Regarding functions, we have determined the attenuation and buffering of the main KEGG superpathways. As can be observed (Supplementary Fig. 6 and Supplementary Fig. 7), a different superpathway response appears for both attenuation and buffering when all age groups are compared. Statistical differences in both attenuation and buffering were detected in six superpathways (Amino Acid Metabolism; Cell Motility; Excretory System; Membrane Transport; Metabolism of Cofactors and Vitamins; Metabolism of Other Amino Acids), three more superpathways showed differences only in attenuation (Cell Growth and Death; Endocrine System; Transport and Catabolism) while another four superpathways were only significant in buffering (Carbohydrate Metabolism; Folding, Sorting and Degradation; Metabolism of Terpenoids and Polyketides; Transcription). The paired Wilcoxon test between age groups confirmed these differences in at least one pair. For attenuation, the most significant differences were observed between groups I-E (8), followed by I-A (5) and A-E (1). For buffering, most significant differences were observed between groups I-A (7), followed by groups A-E (5) and I-E (4).

Considering superpathways, a wide range of attenuation values were recorded, from near -2 to near 5. Superpathways related to universal cellular activities showed attenuation values above 2. Others, which are not universal and associated with a specialized life style show lower values for attenuation. The Metabolism of Terpenoids and Polyketides superpathway is a particular case, with attenuation values near 2.5. This superpathway is involved in reactions related to specialized modes of life, acts on molecules such as terpenoids and carotenoids, and participates in the biosynthesis of antibiotics and siderophores, thereby improving competitiveness within the microbial community.

In all cases, values in superpathways can hide differences in pathways for attenuation and buffering. Therefore, the underlying pathways were also explored (Supplementary Table 9). For attenuation, 54 pathways showed significant differences. In contrast, for buffering, 74 pathways showed significant differences. Paired Wilcoxon tests between age groups confirmed these differences for at least one pair. Interestingly, high values but low differences in amount were found in the superpathway for Amino Acid Metabolism. In contrast, the Tryptophan metabolism pathway (ko00380) showed a low degree of attenuation (I = 0.36 ± 0.35, A = 0.44 ± 0.34, and E = 0.40 ± 0.33), but typical buffering values.

## Discussion

The IAE cohort comprises typically healthy individuals from three age groups whose intestinal microbiota has been studied at different times, ranging from a few months to one or two years in most individuals. First, we observed that the effect of age group is more pronounced in taxa and MG than in MT (Fig. 1; Supplementary Fig. 1). This is understandable if we consider that the expression of bacterial genes across different taxonomic groups responds rapidly and similarly to the metabolic demands imposed by diet or other factors affecting the microbiota, regularly and longitudinally. This result is similar to that of Mehta et al.^27^ in their longitudinal study of a cohort of healthy individuals over 6 months.

Another interesting result, which coincides with those reported^26,27^, is related to the time-course variation in the conserved taxa. According to our evaluation using the Jaccard index, there is taxon conservation within each individual. At short time intervals, the three age groups show a preservation of around 70%, which falls to 60% on average over 1–2 years (Fig. 4). We have also detected interesting differences between the age groups. The Jaccard index for the maximum comparable separation interval between the three groups (approximately 500 days) shows that Adults and Elders hardly drop below 60%. In contrast, Infants drop to 60%, with a higher standard deviation. We also evaluated levels of shared taxa over intervals as long as 80 years for the core taxa Infants, Adults, and Elderly. The most significant conservation of shared taxa over time may also be related to the observation that the parameter *V* of Taylor’s Law is lower in Adults and Elders than in Infants, which points to a more stable microbiota, with a greater number of individuals in those groups within the window of good health^34^ (Fig. 8).

Results on attenuation indicate no important differences between age groups. However, buffering is significantly lower in Adults than in Infants and Elders. Therefore, a hypothetical alteration in the microbial community composition would not imply differences in functional capacity. Still, in Adults, a smaller alteration would be required for functional biases to be observed. Some differences are apparent when comparing superpathways individually. That suggests that differences in responses to alterations in microbial community composition might affect the metabolite landscape, because most superpathways are related to metabolism (particularly Carbohydrate Metabolism; Metabolism of Cofactors and Vitamins; Amino Acid Metabolism; and Metabolism of Other Amino Acids). The study of superpathways and pathways highlights important differences in robustness factors between the superpathway and its included pathways. Concerning Tryptophan metabolism (ko00380), the attenuation values are low and much lower than those observed in the Amino Acid Metabolism superpathway, suggesting a high susceptibility to alterations in taxonomic composition.

As expected for an industrialized, urbanized community, we found a microbiota characterized by abundant members of *Bacteroides*, *Bifidobacterium*, and Firmicutes^35^. The abundance of *Bacteroides* and *Faecalibacterium* has also been detected in other Spanish cohorts^36^. Furthermore, the *Bacteroides* genus has been consistently linked to a westernized lifestyle with animal-based foods, in which protein- and fat-rich diets are abundant^35,37–40^, as is the case for other bile-tolerant microorganisms (*Alistipes*, *Bilophila*, *Parabacteroides*). Moreover, we found some highly prevalent core taxa across the different age groups of our cohort (such as *Bacteroides*, *Faecalibacterium*, *Alistipes*, *Subdoligranulum*, or *Ruminococcus*) that are also highly prevalent in other human populations and lifestyles around the World^40,41^. Previous work shows the presence of core taxa in humans and various non-human primates, including genera such as *Bacteroides*, *Bifidobacterium*, and *Ruminococcus*^42^, suggesting phylogenetic conservation. In addition, a recent study^43^ employing paired phylogenies of humans and their microbiomes revealed parallel evolutionary histories among different species of *Bacteroides*, *Faecalibacterium*, *Anaerostipes*, and *Ruminococcus*, indicating co-diversification with the human host. It has been suggested that *Bacteroides sp*. could be important drivers of the healthy human gut ecosystem^44^. *Bacteroides* are important primary polysaccharide degraders, generating oligosaccharides that secondary degraders can use^45,46^. Furthermore, these species exert important regulatory effects on the host, including accelerating angiogenesis in the intestinal mucosa and enhancing immunity^46^. Likewise, various species of the genus *Ruminococcus* are specialized in breaking down complex polysaccharides in the human gut. They are considered key symbionts, playing important roles in the gut ecosystem^47^.

Age-related changes are also apparent in the prevalence levels of some core taxa, with certain groups consistently becoming more prevalent with age, such as *Blautia*, *Butyricimonas,* and *Coprobacter*. Previous studies also indicate an increased presence and abundance of *Blautia* with age^48–50^. A recent literature review also identified consistent taxa associated with aging^51^. An increase in microbiome taxa, such as *Christensenellaceae* and *Butyricimonas*, has been associated with healthy aging, whereas others, such as *Desulfovibrio*, have been related to unhealthy aging. Furthermore, *Christensenellaceae* has been associated with leaner subjects and better adherence to the Mediterranean diet^52,53^. In contrast, *Coprobacter* has been associated with polypharmacy in the elderly^54^. Therefore, *Blautia*, *Butyricimonas*, and *Coprobacter* could represent age-related markers.

On the other hand, some taxonomic groups, such as *Faecalitalea*, show a more abrupt decrease in prevalence, being core taxa only in childhood. Others, such as *Turicibacter* and *Gordonibacter*, remain core taxa during adulthood but become non-core during elderhood. *Faecalitalea* has been detected as overrepresented in children with respect to adults in other Mediterranean cohorts^55^. Some *Gordonibacter* species can metabolize dietary plant polyphenols into Urolithins^56–58^. These compounds are of particular interest, especially Urolithin-A, since they have immunomodulatory, antioxidant, and anti-inflammatory properties and have been linked to potential benefits in aging^57,58^. *Gordonibacter* is particularly relevant, as to date three species are known to produce urolithins^59,60^. Furthermore, different urolithin metabotypes (UMs) have been described^58–61^, and aging has been reported as a key factor affecting the distribution of UMs within a large Spanish cohort ranging from 5 to 90 years of age^62^, at least for UM-A and UM-B. All this underscores the importance of *Gordonibacter* as a possible marker of healthy aging, as this genus has been associated with Urolithin-A production and the UM-A metabotype and appears inversely correlated with isourolithin-A and Urolithin-B^63,64^.

Finally, we consider the progressive age-related decoupling of some taxa in the human microbiota to be highly relevant. The functional deficits in tryptophan and indole^33^ observed in the elderly were previously supported by directed proteomics and metabolomics. A recent study used animal models to underscore the relevant role of tryptophan and indole in what they call the "quality of life" in old age, based on the fact that their absence was linked to frailty syndromes^65^. In a previous study, we observed that genes involved in tryptophan and indole synthesis had a surprisingly low prevalence in the adult population^66^. We speculate that some bacterial taxa may exert inhibitory effects on others^3,67^ and that the human host could resolve the molecular deficiencies through other pathways. In the case of the TnaA protein, we observe that mRNA synthesis in the genus *Akkermansia* is approximately 10 times lower in Adults and Elders than in Infants, which aligns with the low prevalence or absence of this enzyme in the first two age groups. In another study, the effects of a month-long daily intake of *A. municiphila* on behavior, function, and the redox state of immune cells in old female ICR-CD1 mice (OA group) were evaluated^68^. Compared with age-matched mice that did not receive A. municiphila, the A. municiphila-fed group showed significant improvements in several behavioral responses and an increased lifespan^68^. Supplementation with *A. muciniphila* prevented the age-related decline in the thickness of the colonic mucus layer and attenuated inflammation and immune-related processes in aged mice^69^. In the case of the TrpB protein, we suggest that mRNA synthesis by the genera *Bilophila*, *Enterobacter*, and *Butyricicoccus* is consistent with the low abundance or absence of this enzyme in the Adult and Elderly groups.

Nevertheless, our study has limitations. Firstly, conducting a longitudinal study of such a prolonged duration is a complicated task, which is why it was decided to work with a manageable number of 30 individuals; despite this, not all of them completed the full time series. Furthermore, metatranscriptomics is a field still under development, and it is well known that most protocols are prone to bias toward the most abundant features^70^, which must be considered when interpreting these results.

## Methods

### Cohort recruitment and sampling

We recruited thirty healthy volunteers from the Valencian Region (Spain): ten 2–5 years old Infants (average age 3.9 ± 1.5), ten 27–44 years old Adults (average age 35.4 ± 6.6), and ten 69–81 years old Elders (average age 74.7 ± 4.0) at the first step of the study. The experimental design was scheduled to collect eight fecal samples from each. Finally, out of 240 possible fecal samples, 219 were collected. All volunteers were asked to complete a questionnaire to collect information on their diet, general health, medical history, and antibiotic and other drug use (Supplementary Table 1 and Supplementary Table 2). Individuals with antibiotic prophylaxis between sampling waited at least two weeks after completing their treatment before collecting the next sample. Personal identification was encoded. Each volunteer provided fecal samples according to the researchers’ instructions. Fecal samples were collected in sterile recipients containing 10 mL of RNAlater (Ambion) to stabilize RNA and preserve its integrity.

### DNA and RNA purification

The fecal samples were resuspended in a 50% RNA*late*r™ solution (Invitrogen)/phosphate-buffered saline (PBS), centrifuged to remove fecal debris, and the supernatants were aliquoted. Total DNA was extracted from the pellet using the MagNA Pure LC DNA Isolation kit III (Bacteria, Fungi) (Roche) and the MagNA Pure LC Robot (Roche) for one 500 µL aliquot. A second aliquot was used for total RNA extraction from the pellet using the RNeasy Power Microbiome Kit (Qiagen), and the resulting RNA was subsequently treated with Baseline-ZERO™ DNase (Epicentre). DNA and RNA concentrations were measured with Qubit™ dsDNA HS Assay Kit and Qubit™ RNA HS Assay Kit (ThermoFisher Scientific) in a Qubit 4.0 fluorometer (Invitrogen).

### Amplification and 16S sequencing

From each extracted DNA, the V3-V4 hypervariable regions of the 16S rRNA gene were amplified by PCR with universal forward 5′-TCGTCGGCAGCGTCAGATGTGTATAAGAGACAGCCTACGGGNGGCWGCAG-3′ and reverse primers5′-GTCTCGTGGGCTCGGAGATGTGTATAAGAGACAGGACTACHVGGGTATCTAATCC-3′. Then, Illumina sequencing adapters were attached by PCR using the Nextera XT Index Kit for sequencing. Internal controls of extraction and amplification were also included with the samples. Amplicons were purified using AMPure XP beads (Beckman Coulter), and the quantified libraries were pooled and sequenced on an Illumina MiSeq using 2 × 300 bp.

### Metagenome sequencing

To obtain libraries from the total DNA, the Nextera XT kit (Illumina) was used. The Nextera XT transposase fragmented the input DNA and added adapter sequences to its ends, enabling PCR amplification in subsequent steps. A limited-cycle PCR reaction used these adapter sequences to amplify the insert DNA. PCR products were purified using AMPure XP beads (Beckman Coulter) to clean up the final library. The libraries were quantified using the Qubit™ DNA HS Assay Kit (Thermo Fisher Scientific), and an equimolar library pool was prepared. Sequencing was performed on a MiSeq sequencer, producing 2 × 300-bp reads.

### Metatranscriptome sequencing

First, the abundant rRNA was eliminated to enrich the mRNA samples using the TermoFisher Scientific MICROBExpress™ Kit. Later, libraries were generated following the protocol described in Illumina’s ScriptSeq v2 RNA-Seq library preparation (Epicentre) kit. The resulting libraries were quantified using the Qubit™ DNA HS Assay Kit (Thermo Fisher Scientific), and an equimolar library pool was prepared. Two sequencing batches were generated for the samples: the first was sequenced on the MiSeq system, producing 2 × 300 bp reads, and the second on an Illumina NextSeq 500, producing 1 × 150 bp reads. All age groups were included in each batch, and the majority of individuals were common across batches. Both sets were treated as a single set for the analysis, with this effect taken into account in the subsequent differential analysis.

### Sequence treatment

*16S processing.* Primers were removed using Cutadapt v3.2 with linked behavior, and untrimmed sequences were discarded. Read processing was continued using DADA2 v1.18.0. Quality trimming was performed by truncating forward and reverse reads to 260 and 210 bp, respectively. Low-quality forward and reverse reads were filtered using max expected errors of 2 and 5 (respectively), along with the remaining default parameters. Pool inference behavior was used to determine exact ASVs. Paired-end reads were merged, and chimeric sequences were eliminated using the consensus method. Taxonomy assignation was carried out employing the IDTAXA classifier (DECIPHER v2.18.0) against the SILVA v138 database, with a 50% threshold. ASVs identified as organelles were eliminated. Only ASVs of the Bacteria and Archaea domains were retained. Moreover, ASVs not classified at the phylum level were also filtered. Furthermore, PICRUSt2 v2.4.2 was used to predict functions with the “stratified” and “per_sequence_contrib” parameters. One sample, 16S_A04T7, was discarded due to a suboptimal number of reads.

*MG quality control.* Shotgun reads were quality-trimmed and filtered using Fastp v0.21.0. Adapters were auto-detected and trimmed. Quality trimming was done with Slidingwindow 4:15, Leading 20, Trailing 20, and a global trimming at position 290 bp. Reads containing ambiguous bases (N) or under 60 bp were filtered. Afterward, contaminants (Phix GCF_000819615.1) and host reads (Human GRCh38.p13) were removed using Bowtie2 v2.4.2 and Samtools v1.10. The contaminated region indicated^71^ for GRCh38.p13 was masked using Bedtools v2.29.2. Quality was checked with FastQC v0.11.9 and MultiQC v1.9 at the different steps. Samples that did not reach 100,000 reads were excluded from further analysis.

*MT quality control.* Shotgun reads were quality-trimmed and filtered using Fastp as described for MG data with the following changes: i) For Paired-end files, adapters were auto-detected and trimmed, whereas Nextera adapters were searched and removed for Single-end files; ii) Global trimming was performed for Paired-end and Single-end files at 260 bp and 137 bp, respectively; iii) Reads under 45 bp were filtered. Afterward, contaminants and host reads were removed as described for the MG data. Moreover, rRNA sequences were removed by aligning against the SILVA v138.1 database and 5S and 5.8S rRNA sequences from the Rfam database. Based on Zhang et al.^70^, we decided to work only with MT, with a surviving, quality-controlled MG, and with those that at least had an initial sequencing depth greater than the corresponding MG. Samples that did not reach 90,000 reads were excluded from further analysis.

*Functional profiling.* MG and MT data were processed in merged mode using the Squeezemeta pipeline v1.5.2 for functional profiling, with the “singletons” parameter set to 0 (no binning). Internally, reads were assembled into contigs using Megahit. Similarity searches for GenBank, eggNOG, and KEGG were performed. The normalization method for MG and MT annotation was RPKM (reads per kilobase of transcript per million mapped reads).

### Statistical analysis of the microbiome

For both 16S and MG/MT, repeated measures were accounted for, with a random effect included in the models. Additionally, a fixed effect for MT was included to account for possible batch effects. We used the ANCOMBC_2.0.2 model for the 16S case, and the linear mixed model implemented in the R library nlme for the MG/MT. Volcano plots were constructed using ‘EnhancedVolcano’ v1.14.0. Alpha diversity was calculated for the absolute abundances of 16S data using the Chao1 estimator and the Shannon index. The diversity between samples (beta diversity) was assessed with Permanova using the Bray–Curtis dissimilarity index, previously obtained from ANCOMBC-corrected taxa compositions for 16S data and from KOs’ relative abundances for MG and MT. Alpha and beta diversity were computed using vegan v2.5–7.

*Core microbiome analysis.* The core analysis was carried out using the Microbiome package. Cores were defined at various prevalence levels (100%, 80%, and 50%) for the different age groups with a 1e − 4 relative abundance threshold. Intersections between cores were compared using ggvenn and ggplot2. Abundance and prevalence were also examined with ComplexHeatmap v2.12.0.

*Jaccard measurements of changing diversity with higher time intervals.* The Jaccard indices were calculated using the jacpop package. The associated graphs were generated with Python 3 using matplotlib. To study how taxon conservation of the microbiome changes with aging, a modification of the Jaccard similarity index, the Jaccard core index, was defined. These indices were calculated by taking the 50% prevalence core taxa of one of the age groups as a fixed reference. For this purpose, Jaccard’s index was calculated for each sample as (*Number of common taxa*) / [(*Number of core taxa*) + (*Number of taxa in sample*)—(*Number of common taxa*)]. A 1e − 4 relative abundance threshold was applied.

*Stability analyses.* The stability of the gut microbiota has been assessed using the model developed^34^, based on Taylor’s power law, which characterizes the microbiota’s stability for a given individual across several time points. Two fundamental parameters can be estimated: the variance of the abundance of each taxon (*V*) and the coefficient of the power law (*β*), which correspond to the intercept and the power law slope, respectively. Both parameters appear to be correlated with community stability and can serve as proxies.

On the one hand, β is a scaling index that provides information about the ecosystem’s statistical properties. If it is 1/2, the system behaves like a Poisson distribution. If *β* is 1, the system follows an exponential distribution. Metagenomes generally undergo time-course variations with β between these two universal classes. On the other hand, *V* is a direct estimator of the amplitude of fluctuations over time. *V* represents the maximum variability attainable by a hypothetically dominant taxon (genus in our case), with relative abundance close to 1. *V* is an important parameter that characterizes the system type. If *V* is small, the ranking is stable; if *V* becomes larger, it indicates an increasingly unstable metagenome.

*Taxa-function robustness.* The microbiome’s functional capacities depend on the microbial community’s taxonomic structure, because each taxon harbors specific functions at particular abundances. Then, the metagenome is determined by the taxonomic composition of the microbial community and the abundance of each one, and changes in its composition and abundance of one or more taxa lead to changes in functional capacities. These have recently been described as taxa-function or species-function relationships.

To determine changes in functional capacities or, more formally, to quantify the number of changes in gene composition induced by changes in taxonomical structure, Eng and Borenstein^72^ described an approach to evaluate the taxa-function robustness and quantify two main factors. The first is attenuation, or the rate at which the functional shift increases with increasing perturbation magnitude. The second, buffering, is conceptually defined as the size of a taxonomic perturbation required for noticeable functional shifts. These two factors can be measured globally or for each superpathway or pathway, thereby indicating the weakest points in the global microbiota metabolism, where stochastic changes in the microbial community, including dysbiosis, can result in significant deviations in the functional profile. The robustness parameters were determined using the software available at https://github.com/borenstein-lab/robustness. However, some pipeline modifications were implemented to improve sensitivity and accuracy. First, using Picrust2, a table of 16S rRNA gene copy numbers, a genomic content annotation table, and a phylogenetic tree were generated; second, these three files were used to replace the files provided by the original pipeline. Various data treatments, graphical representations, and statistical tests were performed using dplyr, ggplot2, and ggpubr.

## Supplementary Information

Supplementary Information 1.

Supplementary Information 2.

Supplementary Information 3.

Supplementary Information 4.

Supplementary Information 5.

Supplementary Information 6.

Supplementary Information 7.

Supplementary Information 8.

Supplementary Information 9.

Supplementary Information 10.

Supplementary Information 11.

Supplementary Information 12.

Supplementary Information 13.

Supplementary Information 14.

Supplementary Information 15.

Supplementary Information 16.

Supplementary Information 17.

## Data Availability

The sequences from the 16S rDNA gene, MG, and MT, as well as, metadata were deposited in the EBI Short Read Archive under the study accession number PRJEB39661 (https://www.ebi.ac.uk/ena/browser/view/PRJEB39661), with accession numbers ERS4861276-ERS4861494 for 16S rDNA gene, ERS4861057-ERS4861275 for MG and ERS4861495-ERS4861579 for MT, respectively, and under the study accession number PRJEB62571 (https://www.ebi.ac.uk/ena/browser/view/PRJEB62571), with accession numbers ERS15546495-ERS15546553 for MT. Correspondence between the submitted files and the final selection after quality control, by data type, is shown in Supplementary Table S10. Scripts used for the different analyses can be found at GitHub (https://github.com/tbcgit/IAE_Cohort).
