# Supplementary material for: Lifetime existence of a core of mutualistic symbionts and functionally uncoupled taxa in the gut of a Mediterranean cohort
Source: Sci Rep. 2026 Jan 9;16:4921. doi: 10.1038/s41598-026-35033-3 (PMC12873169; doi:10.1038/s41598-026-35033-3)
Supplement: Supplementary file 1 — Supplementary Information 1. [file 41598_2026_35033_MOESM1_ESM.pdf]

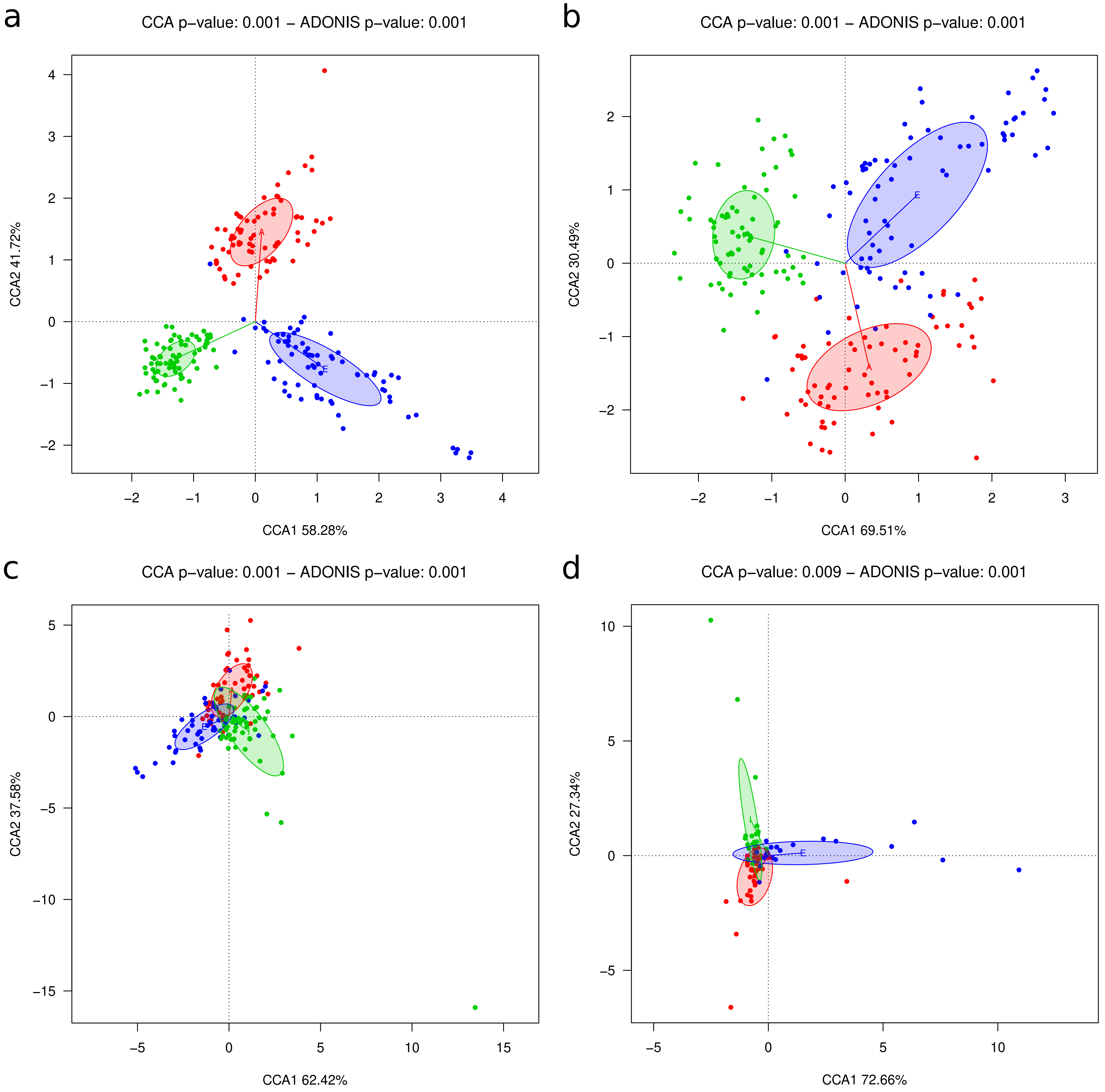


**Figure S1.** CCA analysis. CCA based on 16S rRNA gene taxonomy, including ASVs (a) and genus (b) levels, as well as functions from whole genome shotgun MG (c) and MT (d). Significance was set with p−value <= 0.05.
