# Supplementary material for: Lifetime existence of a core of mutualistic symbionts and functionally uncoupled taxa in the gut of a Mediterranean cohort
Source: Sci Rep. 2026 Jan 9;16:4921. doi: 10.1038/s41598-026-35033-3 (PMC12873169; doi:10.1038/s41598-026-35033-3)
Supplement: Supplementary file 2 — Supplementary Information 2. [file 41598_2026_35033_MOESM2_ESM.pdf]

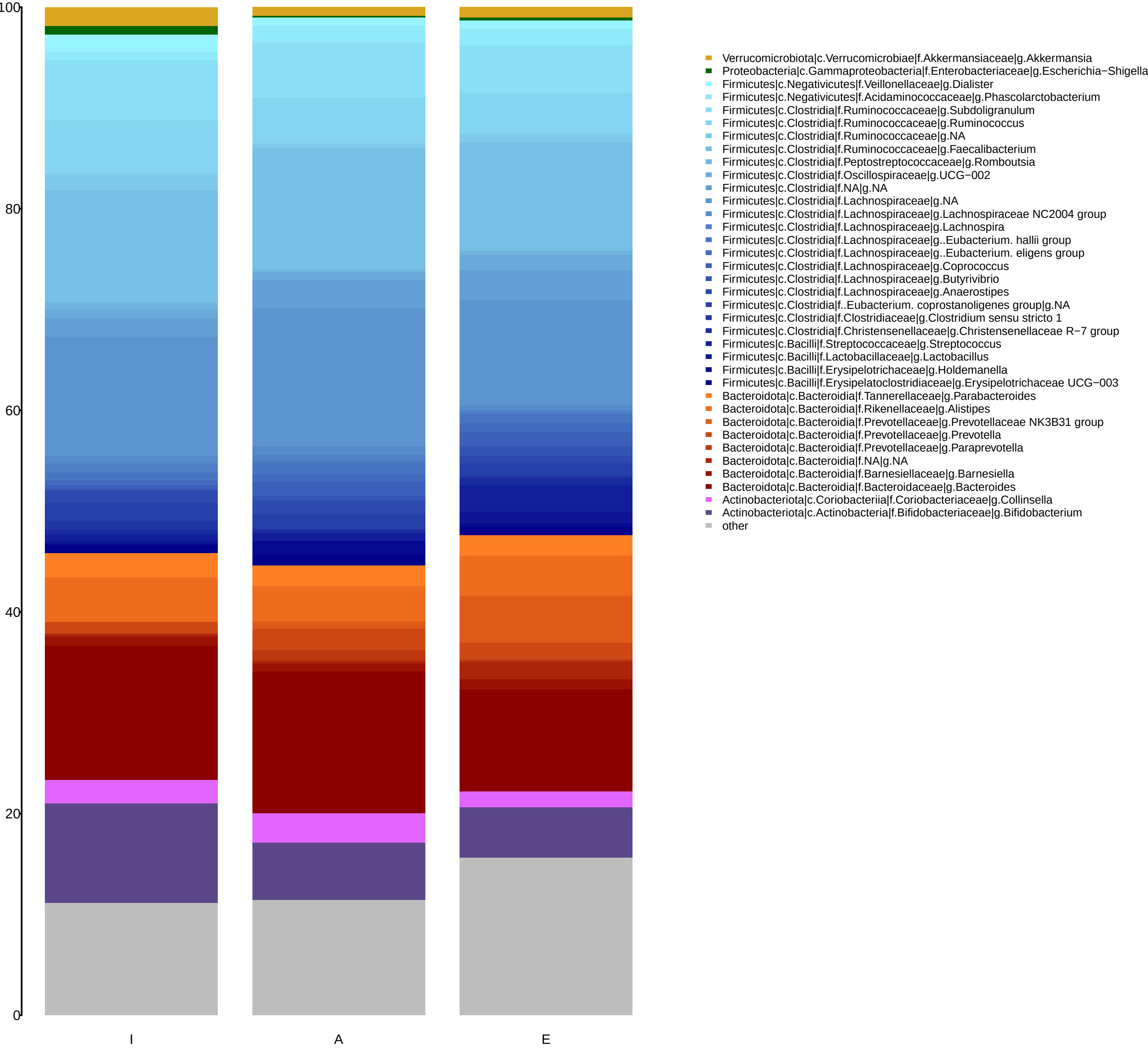

Figure S2. Microbiome composition barplots based on 16S rRNA gene. Average taxa composition per age group is represented.
