# Supplementary material for: Lifetime existence of a core of mutualistic symbionts and functionally uncoupled taxa in the gut of a Mediterranean cohort
Source: Sci Rep. 2026 Jan 9;16:4921. doi: 10.1038/s41598-026-35033-3 (PMC12873169; doi:10.1038/s41598-026-35033-3)
Supplement: Supplementary file 3 — Supplementary Information 3. [file 41598_2026_35033_MOESM3_ESM.pdf]

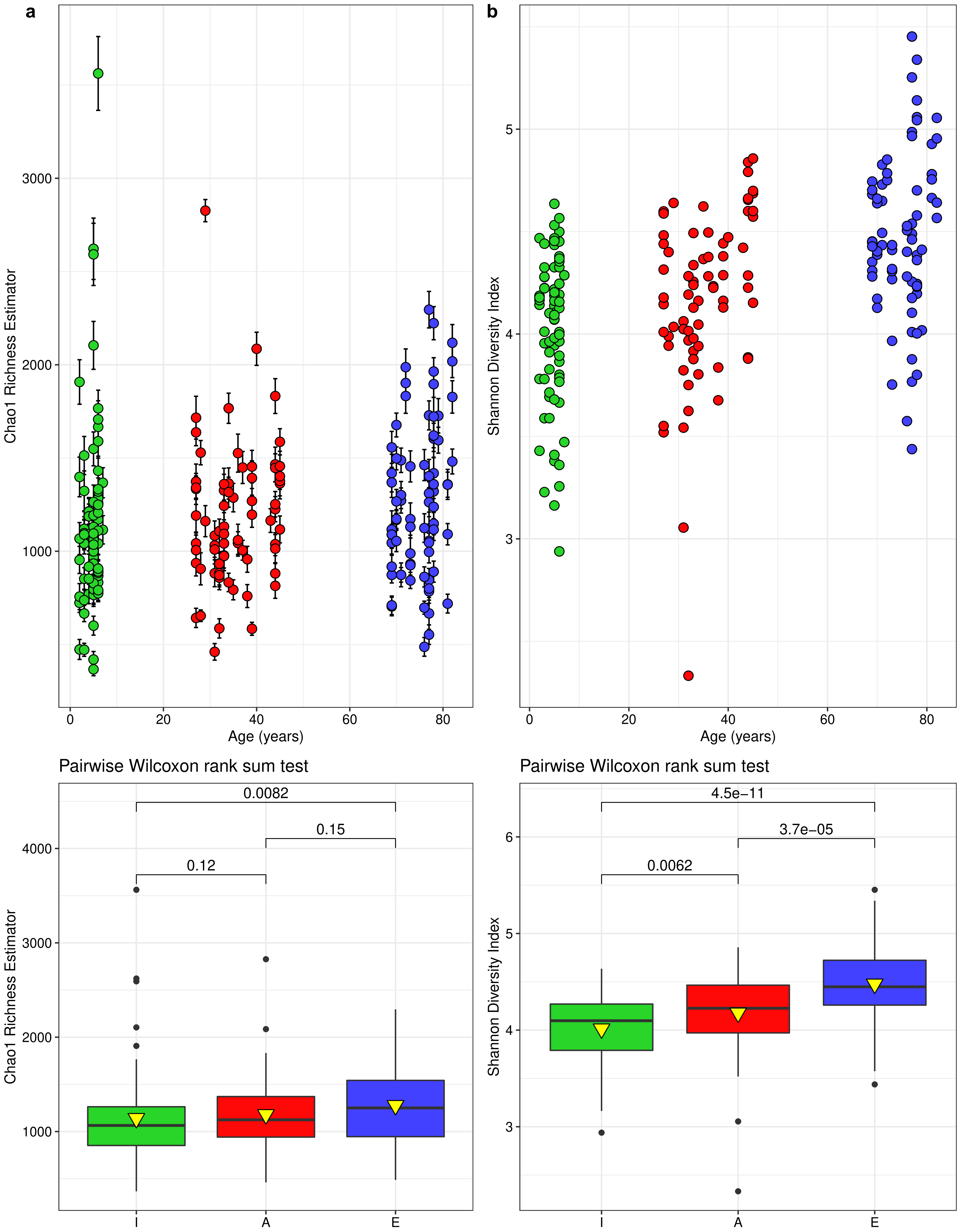

Figure S3. Alpha diversity metrics based on ASVs 16S rRNA gene. Chao1 Richness Estimator (a) and Shannon Diversity Index (b) were calculated. The top panels present the alpha diversity metrics as a function of age. The standard error is also represented in the case of Chao1 Richness Estimators. Bottom boxplot panels represent comparisons between age groups using the Wilcoxon rank sum test. In the boxplots, the black line within the box marks the median, and the yellow triangle the mean. Significance was set with p−value <= 0.05.
