# Supplementary figures and images for: Lifetime existence of a core of mutualistic symbionts and functionally uncoupled taxa in the gut of a Mediterranean cohort
Source: Sci Rep. 2026 Jan 9;16:4921. doi: 10.1038/s41598-026-35033-3 (PMC12873169; doi:10.1038/s41598-026-35033-3)

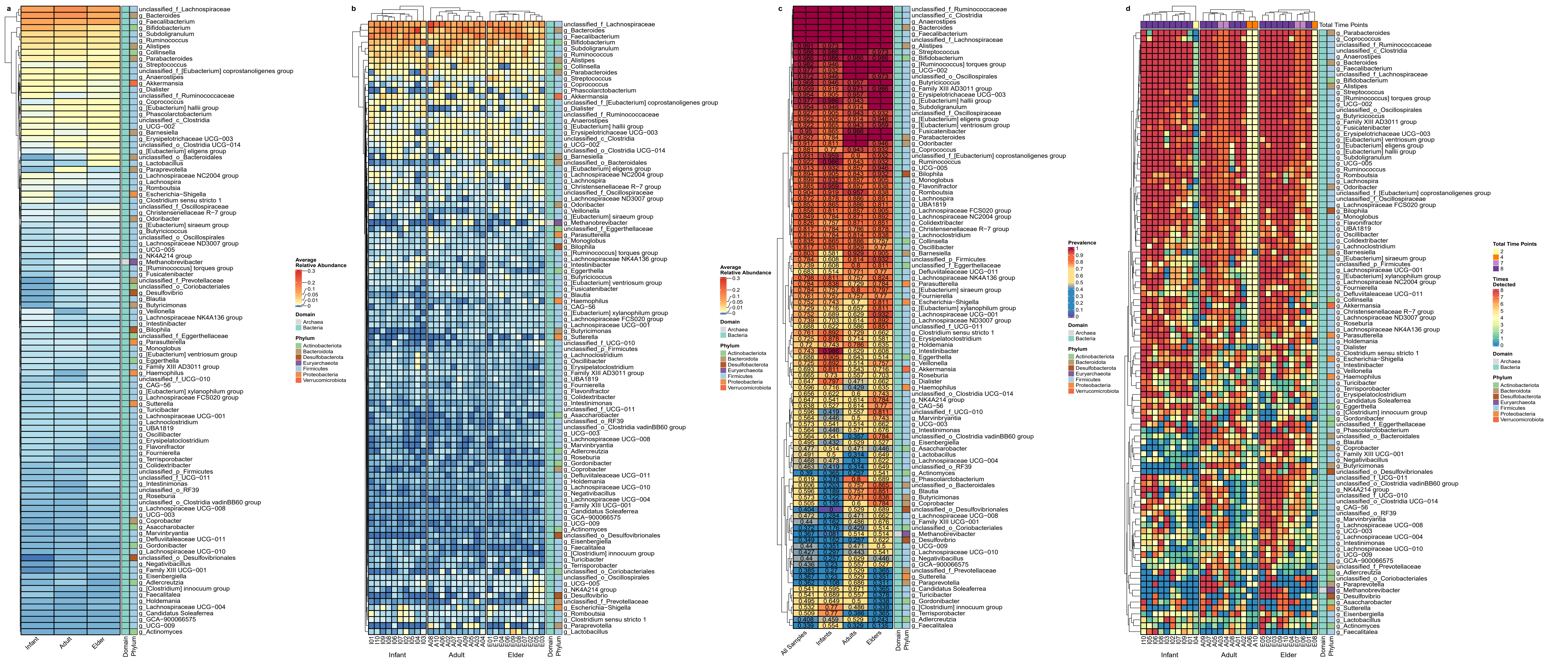

Supplement: Supplementary file 4 — Supplementary Information 4. [file 41598_2026_35033_MOESM4_ESM.pdf]
