# Supplementary material for: Lifetime existence of a core of mutualistic symbionts and functionally uncoupled taxa in the gut of a Mediterranean cohort
Source: Sci Rep. 2026 Jan 9;16:4921. doi: 10.1038/s41598-026-35033-3 (PMC12873169; doi:10.1038/s41598-026-35033-3)
Supplement: Supplementary file 5 — Supplementary Information 5. [file 41598_2026_35033_MOESM5_ESM.pdf]

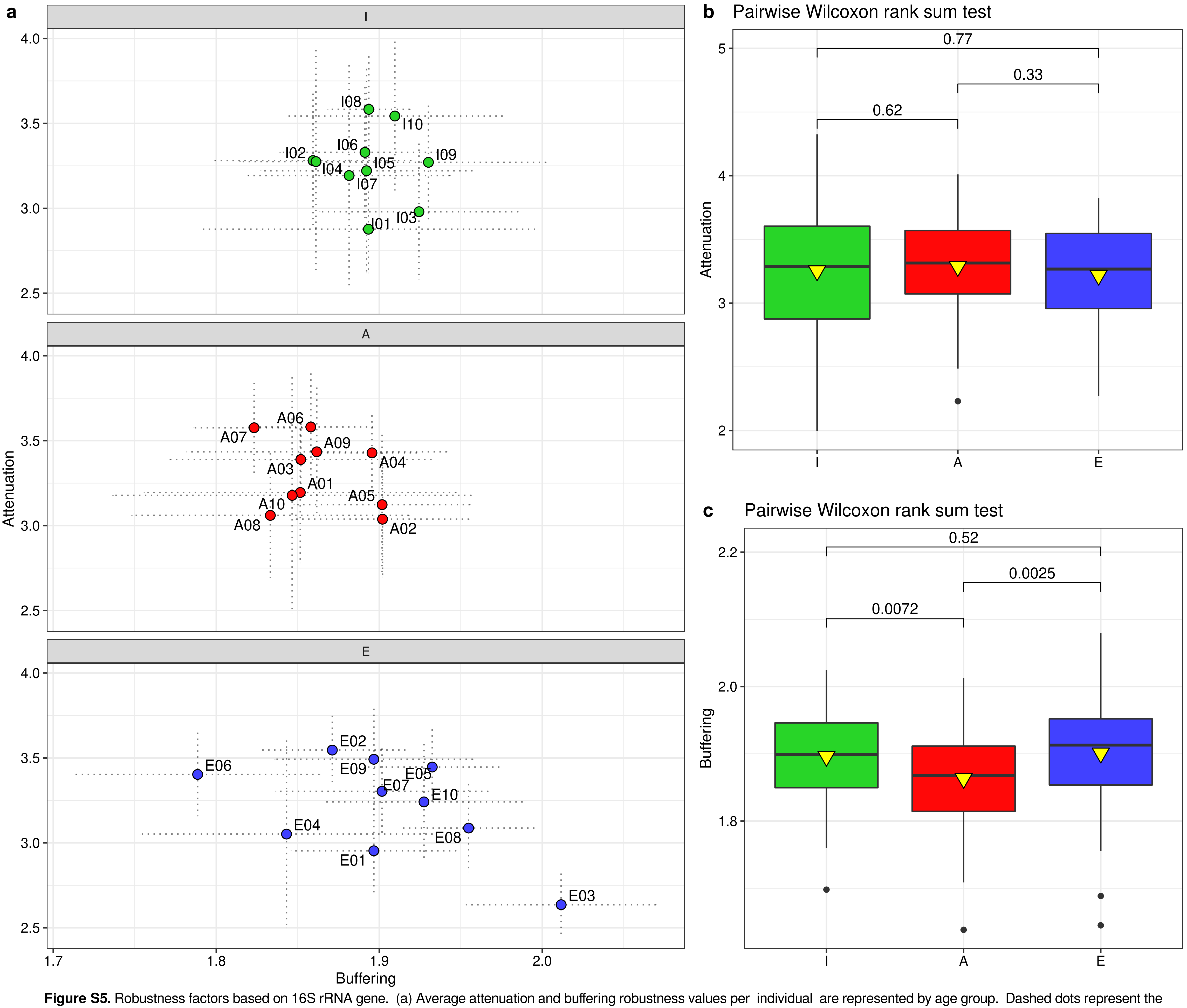

Figure S5. Robustness factors based on 16S rRNA gene. (a) Average attenuation and buffering robustness values per individual are represented by age group. Dashed dots represent the standard deviation per individual. (b) Pairwise comparisons for attenuation between age groups using the Wilcoxon rank sum test are also represented. (c) Pairwise comparisons for buffering between age groups using the Wilcoxon rank sum test are also represented. In the boxplots, the black line within the box marks the median, and the yellow triangle the mean. Significance was set with p−value <= 0.05.
