# Supplementary material for: Lifetime existence of a core of mutualistic symbionts and functionally uncoupled taxa in the gut of a Mediterranean cohort
Source: Sci Rep. 2026 Jan 9;16:4921. doi: 10.1038/s41598-026-35033-3 (PMC12873169; doi:10.1038/s41598-026-35033-3)
Supplement: Supplementary file 6 — Supplementary Information 6. [file 41598_2026_35033_MOESM6_ESM.pdf]

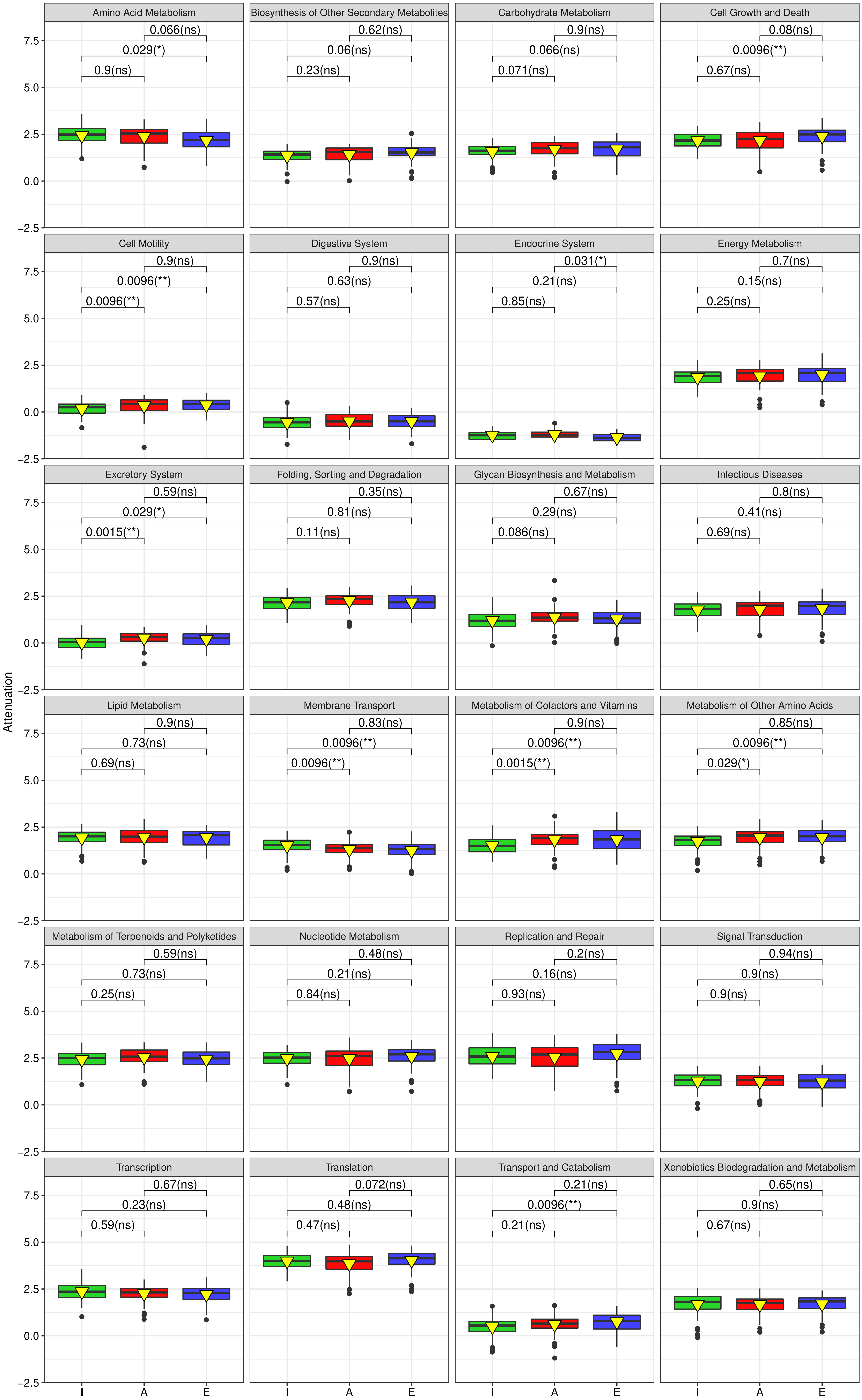

Figure S6. Robustness attenuation values per superpathway based on 16S rRNA gene. Boxplot panels represent comparisons between age groups using the Wilcoxon rank sum test. In the boxplots, the black line within the box marks the median and the yellow triangle the mean. Adjusted p-values were corrected with the Benjamini-Hochberg method. Different adjusted p-value cutpoints are represented (ns:1, *:0.05, **:0.01, ***:0.001, ****:1e-04). Significance was set with adjusted p-value <= 0.05.
